# Aβ Induces Excitotoxicity Mediated by APC/C-Cdh1 Depletion That Can Be Prevented by Glutaminase Inhibition Promoting Neuronal Survival

**DOI:** 10.1038/srep31158

**Published:** 2016-08-12

**Authors:** T. Fuchsberger, S. Martínez-Bellver, E. Giraldo, V. Teruel-Martí, A. Lloret, J. Viña

**Affiliations:** 1 Department of Physiology, Faculty of Medicine, University of Valencia, INCLIVA Avda. Blasco Ibañez 15, 46010 Valencia, Spain; 2 Department of Anatomy and Human Embriology, Faculty of Medicine, University of Valencia, Avda. Blasco Ibañez 15, 46010 Valencia, Spain; 3 Department of Cellular Biology and Parasitology, Faculty of Biology, University of Valencia, Avda. Doctor Moliner 50, 46100 Valencia, Spain

## Abstract

The E3 ubiquitin ligase anaphase-promoting complex/cyclosome (APC/C) is activated by the fizzy-related protein homolog/CDC20-like protein 1 (cdh1) in post-mitotic neurons. Growing evidence suggests that dysregulation of APC/C-Cdh1 is involved in neurodegenerative diseases. Here we show in neurons that oligomers of amyloid beta (Aβ), a peptide related to Alzheimer’s disease, cause proteasome-dependent degradation of cdh1. This leads to a subsequent increase in glutaminase (a degradation target of APC/C-Cdh1), which causes an elevation of glutamate levels and further intraneuronal Ca^2+^ dysregulation, resulting in neuronal apoptosis. Glutaminase inhibition prevents glutamate excitotoxicity and apoptosis in Aβ treated neurons. Furthermore, glutamate also decreases cdh1 and leads to accumulation of glutaminase, suggesting that there may be a positive feedback loop of cdh1 inactivation. We confirmed the main findings *in vivo* using microinjection of either Aβ or glutamate in the CA1 region of the rat hippocampus. We show here for the first time *in vivo* that both Aβ and glutamate cause nuclear exclusion of cdh1 and an increase in glutaminase. These results show that maintaining normal APC/C-Cdh1 activity may be a useful target in Alzheimer’s disease treatment.

The anaphase promoting complex/cyclosome (APC/C) is a large protein complex forming an E3 RING finger ubiquitin ligase that has a canonical role as a cell cycle regulator in proliferating cells^1^,^2^. Gieffers *et al*.^3^ first described a role for APC/C is the nervous system, showing that cdh1, an activator subunit of APC/C, is expressed in post-mitotic neurons^4^. Since then, functions of APC/C in several major processes in the nervous system have been discovered. It was shown that APC/C-Cdh1 controls neuronal G0 maintenance^3^, axonal growth^5^, coordinates neurogenesis^6^, synaptic plasticity^7^, and APC/C-Cdc20 has implications in dendrite morphogenesis and in presynaptic differentiation^8^,^9^. Cdh1 is regulated by phosphorylation, catalyzed by cdk5 in neurons^10^. Non-phosphorylated cdh1 is localized in the cellular nucleus, activating APC/C. When it becomes phosphorylated, cdh1 is translocated to the cytoplasm and degraded^3^,^11^,^12^.

APC/C-Cdh1 recognizes proteins by specific amino acid motifs like KEN-BOX or D-BOX sequences and targets them for degradation. An aberrant accumulation of some of these proteins has been associated with neurodegeneration^13^. An increase in the APC/C-Cdh1 target cyclin B1^10^ has been related to the re-entrance into an ectopic cell cycle in neurons, a phenomenon which we observed in Alzheimer’s disease (AD)^14^. In this work we tested the hypothesis that APC/C could have a relevant pathophysiological role in AD.

We analyzed the effect of oligomeric Aβ_1-42_, a toxic peptide abundantly present in AD brains, on cdh1 *in vitro* and *in vivo.* Furthermore, we tested the role of glutamate on APC/C-Cdh1 activity in neuron culture, since it has been reported that glutamate excitotoxicity dysregulates cdh1^10^. Moreover, increased glutamate levels in the cerebrospinal fluid have been related to AD^15^,^16^. In our experimental setups we also studied the role of glutaminase, which was identified as a target of APC/C-Cdh1^17^. This enzyme has important functions in neurons, as it converts glutamine to glutamate and ammonia. Glutamate is the most abundant neurotransmitter in the brain and has a wide range of functions. One of these is the activation of N-methyl-D-aspartate (NMDA) receptors which causes an intracellular Ca^2+^ increase in neurons. Dysregulation of Ca^2+^ homeostasis impairs mitochondrial oxidation, increases cdk5-p25 levels, and leads to hyperphosphorylation of tau^18^,^19^.

We show here *in vitro* (in primary culture of neurons) and *in vivo* (by microinjections in the hippocampal CA1) that Aβ lowers cdh1 levels in the nucleus. This is mediated by cdk5-p25. Low APC/C-Cdh1 activity results in an increase in glutaminase levels which leads to increased glutamate generation. We have also found that Aβ- or glutamate-induced apoptosis can be ameliorated by inhibition of glutaminase. In addition we observed alterations of cdh1 and glutaminase in the APP/PS1 mouse model of AD. These results provide a molecular mechanism that contributes to glutamate excitotoxicity in AD, mediated by inhibition of APC/C-Cdh1.

## Results

### Aβ induces a proteasome-dependent degradation of cdh1

**Figure 1:**
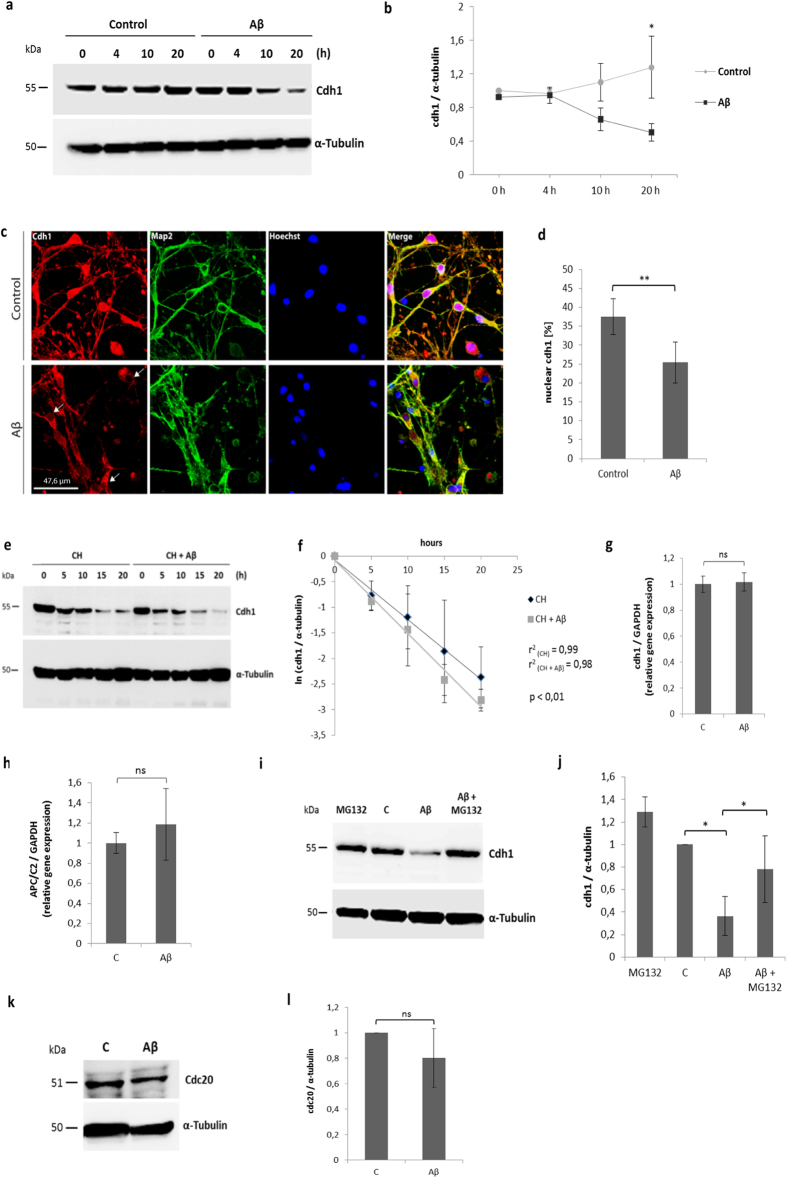
Cdh1 decreases upon Aβ treatment in neurons. **(a)** Representative Western blot image of cdh1 after different time points in control conditions or with Aβ treatment. **(b)** Blots of three independent experiments were quantified by densitometry and normalized against α-tubulin; the mean values ± SD are shown and are statistically significant after 20 h of treatment (p < 0,05). **(c)** Confocal microscope analysis of neurons shows cdh1 (red), neuronal marker map2 (green) and nuclei stain with hoechst (blue). Representative images of three independent experiments show a decrease of cdh1 in nuclei (indicated by arrows). **(d)** Mean values ± SD of nuclear cdh1 are shown in the histogram (p < 0,01). **(e)** Representative Western blot image of cdh1 at different time points after treatment with cycloheximide (CH) or CH + Aβ. **(f)** Blots of three independent experiments were quantified by densitometry and normalized against α-tubulin; the mean values ± SD are indicated. The standard errors of the slopes are: SE (C) = 0,0056; SE (Aβ) = 0,0098. The r square coefficients are indicated in the figure. The results are statistically significant (p < 0,01). (**g,h)** Mean values ± SD of mRNA levels of cdh1 and APC/C2 normalized against GAPDH in control conditions or with Aβ treatment for 24 h are shown. **(i)** Representative Western blot image of cdh1 protein in neurons upon treatment with MG132, in control conditions, with Aβ treatment or with Aβ + MG132. **(j)** Blots of three independent experiments were quantified by densitometry and normalized against α-tubulin; the mean values ± SD are indicated (p < 0,05; p < 0,05). **(k)** Western blot image of cdc20 after 24 hours in control conditions or with Aβ treatment in neurons. **(l)** Blots of three independent experiments were quantified by densitometry and normalized against α-tubulin; the mean value ± SD is shown.

Neurons were incubated with Aβ oligomers (5 μM) for 0 h, 4 h, 10 h and 20 h, and the samples were subjected to Western blot analysis. We detected slightly lower cdh1 levels after 10 h and a statistically significant decrease in cdh1 protein levels after 20 h of treatment (Fig. 1a,b). Immunocytochemical analysis of neurons, stained with map2, showed a significant decrease of cdh1 in the nucleus when treated with Aβ (Fig. 1c,d).

We tested whether the protein half-life of cdh1 changed with Aβ treatment. Therefore we measured cdh1 protein level when protein synthesis was inhibited using cycloheximide (CH) alone or in the presence of Aβ. We calculated the protein half-life of cdh1 which was reduced from 5,9 h to 4,8 h when treated with Aβ (Fig. 1e,f). To further test whether Aβ-induced decrease of cdh1 is caused only by its degradation or whether it also depends on changes in gene expression, we measured mRNA levels of cdh1 and APC/C2, the catalytic subunit of the protein complex, using qPCR. Aβ did not induce any significant changes in their expression level (Fig. 1g,h).

Next, we tested whether the Aβ-induced decrease in cdh1 was dependent on proteasome degradation. Neurons were treated with Aβ alone or with Aβ and the proteasome inhibitor MG132, and we observed that the cdh1 decrease upon Aβ treatment was prevented when the proteasome was inhibited (Fig. 1i,j).

We also tested the protein levels of cdc20, another co-activator subunit of APC/C, in control conditions and upon Aβ treatment. There was a trend toward cdc20 decrease upon Aβ treatment, which was however not statistically significant (Fig. 1k,l).

### Aβ induces an increase in glutaminase, glutamate and apoptosis

**Figure 2:**
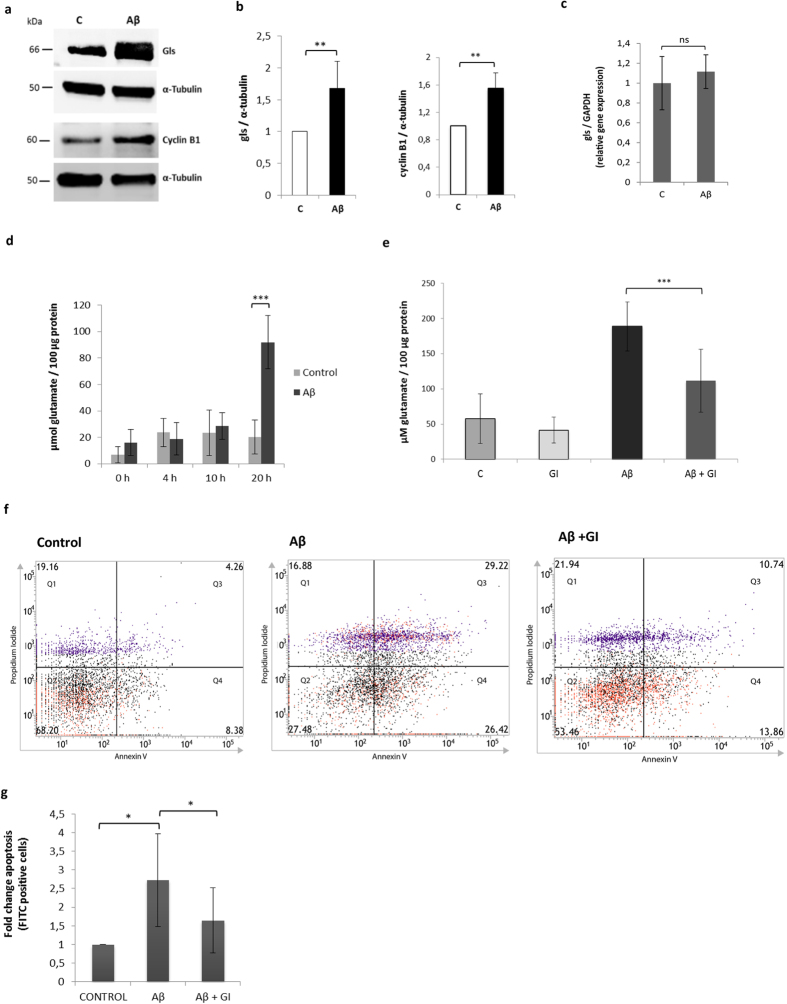
Glutaminase (gls) increases upon Aβ treatment causing elevated glutamate levels and apoptosis. **(a)** Western blot analysis of cyclin B1 and glutaminase after incubation with Aβ for 20 h compared to control conditions. **(b)** Blots of three independent experiments were quantified by densitometry and normalized against α-tubulin; the mean values ± SD are shown (p < 0,01; p < 0,01). **(c)** Mean values ± SD of mRNA levels of glutaminase normalized to GAPDH in control conditions or with Aβ treatment for 20 h are shown. **(d)** Glutamate levels in control conditions or with Aβ for 0, 4, 10 or 20 h in the extracellular culture medium of neurons. Mean values ± SD of glutamate normalized to 100 μg protein of six independent experiments are shown (p < 0,001). **(e)** Mean values ± SD of glutamate normalized to 100 μg protein of six independent experiments after treatment with a glutaminase inhibitor (GI), Aβ or Aβ + GI, (p < 0,001) for 30 h are shown. **(f)** Representative assay of flow cytometry analysis for apoptosis of neurons under control conditions, treated with Aβ or Aβ + GI for 20 h. **(g)** Three independent experiments were quantified and fold change ± SD of apoptotic cells (Q3, Q4) is shown in the histogram (p < 0,05; p < 0,05).

We tested whether the protein levels of APC/C-Cdh1 degradation targets changed when neurons were treated with Aβ, and we observed an increase in cyclin B1 and glutaminase after 20 h of treatment (Fig. 2a,b). We measured the mRNA expression level of glutaminase in neurons under control conditions or treated with Aβ, and did not detect any significant differences (Fig. 2c).

Then, we measured the concentration of glutamate in the culture medium of neurons under control conditions and upon Aβ treatment at 0, 4, 10 and 20 h. We detected a highly significant increase in glutamate levels after 20 h of treatment with Aβ (Fig. 2d). This increase in glutamate was partially prevented when benzophenanthridinone (100 μM), a cell-permeable allosteric glutaminase inhibitor (GI), was added to the Aβ treated cells (Fig. 2e), suggesting that the mode of action may be via glutaminase.

We measured apoptosis levels by flow cytometry in neurons under control conditions or when treated with Aβ or with Aβ + GI. We observed that Aβ induced a two-fold increase in apoptosis, which was almost completely abolished when neurons were treated with GI at the same time (Fig. 2f,g). GI treatment alone had no effect on cell viability or apoptosis (Supl. Fig. 1).

### Inhibition of APC/C leads to an increase in glutaminase and glutamate levels and induces apoptosis

**Figure 3:**
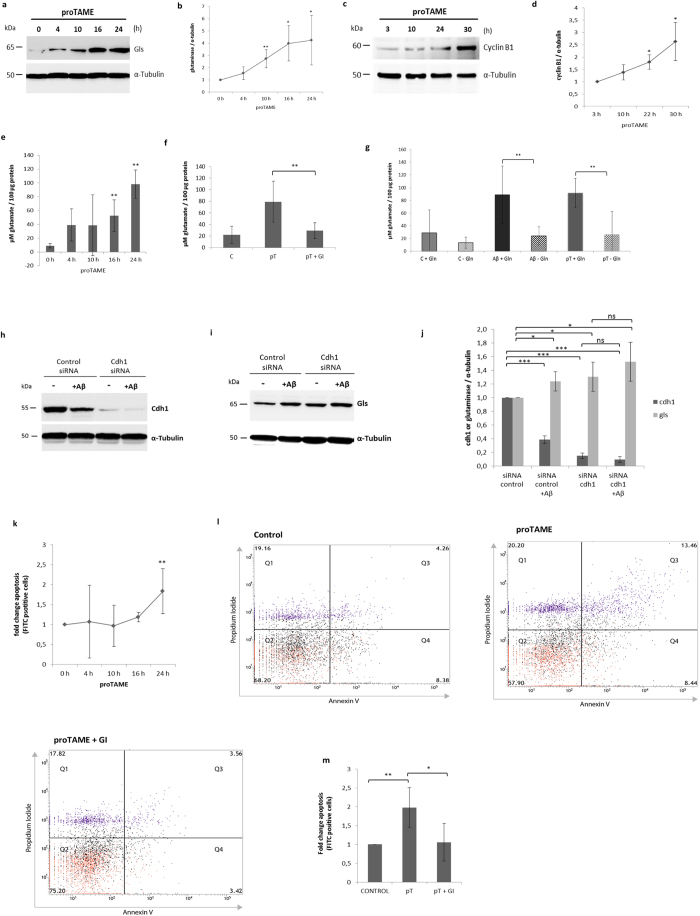
Inhibition of APC/C causes an increase in glutaminase, glutamate and apoptosis. **(a–d)** Western blot images of glutaminase (gls) and cyclin B1 in neurons at different time points with proTAME (pT) treatment (12 μM). Blots were quantified by densitometry and normalized against α-tubulin levels; mean values ± SD of three independent experiments are shown and are significant after 10 h (p < 0,01), 16 and 24 h (p < 0,05) for glutaminase. Cyclin B1 levels are significant after 22 and 30 h (p < 0,05) compared to 3 h treatment with pT. **(e)** Mean values ± SD of glutamate levels after 0, 4, 10, 16 and 24 h with pT (12 μM) of three independent experiments (p < 0,01). **(f)** Mean values ± SD of glutamate levels in controls, or with pT, or pT + glutaminase inhibitor (GI) for 24 h of three independent experiments (p < 0,01). **(g)** Mean values ± SD of glutamate levels in controls in standard medium (SM) containing glutamine (gln) (C + Gln), without glutamine (C − Gln), in SM treated with Aβ (Aβ + Gln) or in medium without gln treated with Aβ (Aβ − Gln) (p < 0,01), in SM treated with pT (pT + Gln) or in medium without gln treated with pT (pT − Gln) for 20 h, of five independent experiments (p < 0,01). **(h–j)** Western blot of cdh1 and gls with control siRNA, or with cdh1 siRNA, each with or without Aβ. The blots were quantified by densitometry and normalized against α-tubulin; mean ± SD of three independent experiments are shown (p < 0,001; p < 0,05). **(k–m)** Flow cytometry analysis for apoptosis in neurons. **(k)** Mean fold change ± SD of three independent experiments of apoptotic cells upon a time-course of pT treatment at 0, 4, 10, 16 or 24 h is shown (p < 0,01). **(l,m)** Representative assays of controls, or treated with pT or pT + GI for 24 h. Three independent experiments were quantified and the fold change ± SD of apoptotic cells (Q3, Q4) is shown (p < 0,01; p < 0,05).

Neurons in culture were treated with proTAME (12 μM), a cell permeable inhibitor of APC/C activity, for 0, 4, 10, 16 and 24 h. Glutaminase increased significantly after 10, 16 and 24 h of treatment (Fig. 3a,b). As expected, inhibition of APC/C also led to an accumulation of cyclin B1 (Fig. 3c,d). Furthermore, the concentration of glutamate in the extracellular medium of neurons treated with proTAME was significantly elevated after 16 and 24 h (Fig. 3). When glutaminase was inhibited in proTAME treated neurons, glutamate did not increase (Fig. 3f). Moreover, the Aβ or proTAME**-**induced increase in glutamate was completely abolished when glutamine was removed from the culture medium (Fig. 3g).

We then tested whether cdh1 depletion, using cdh1 siRNA, is sufficient to induce glutaminase accumulation. We observed that cdh1-silencing (efficiency of about 85%), significantly increases glutaminase, compared to control siRNA treated neurons (Fig. 3h–j). Furthermore, we tested whether Aβ treatment of cdh1-silenced neurons causes an additional increase in glutaminase levels. The Aβ treatment, which further caused a slight decrease of cdh1 (90% cdh1 depletion), induced a trend towards glutaminase increase compared to control siRNA or Aβ-treated neurons alone, which was however not significant (Fig. 3h).

We used flow cytometry analysis to determine whether inhibition of APC/C activity induced apoptosis in neurons. They were treated with proTAME for 0, 4, 10, 16 and 24 h and we observed a significant increase in apoptosis upon the treatment after 24 h (Fig. 3k; Suppl. Fig. 2). When glutaminase was inhibited in proTAME treated neurons, apoptosis was significantly lowered (Fig. 3l,m).

### Cdk5 mediates cdh1 decrease after Aβ or glutamate treatment

**Figure 4:**
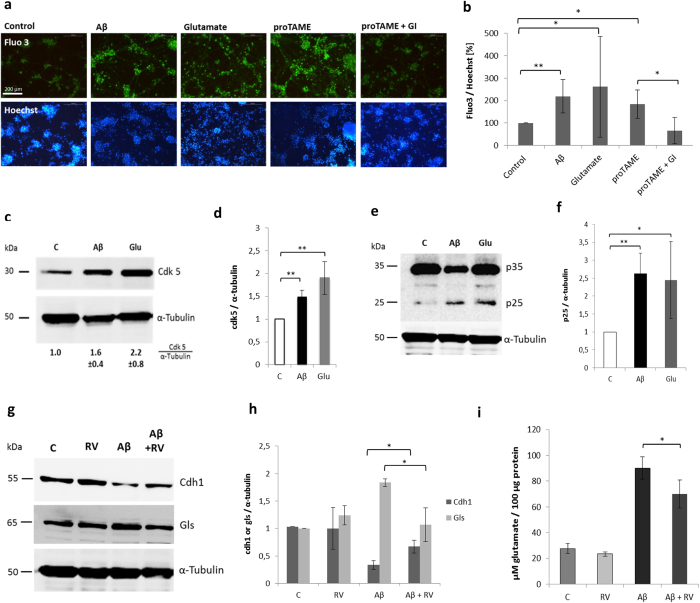
Aβ-induced cdh1 decrease is mediated by Ca^2+^ and cdk5-p25. **(a)** Representative images of Ca^**2+**^ levels by Fluo4 in neurons upon different treatments C, Aβ (5 μM), glutamate (500 μM), proTAME (12 μM), proTAME (12 μM) + GI (100 μM) for 24 h. Ca^**2+**^ intensities (fluo4) were normalized to corresponding nuclei staining (Hoechst). The mean values ± SD from 10 images of each of three independent experiments are shown (p_[C; Aβ]_ < 0,01; p_[C; Glut]_ < 0,05; p_[C; pT]_ < 0,05; p_[pT; pT_ + _GI]_ < 0,05). **(c–f)** Western blots show cdk5 or p25/p35 in neurons under control conditions, with Aβ or glutamate treatment. Blots of three independent experiments were quantified by densitometry and normalized against α-tubulin levels. The mean values ± SD are indicated of cdk5 (p < 0,01, p < 0,01) and p25 (p < 0,01, p < 0,05). **(g)** Western blot image shows cdh1 and glutaminase (gls) in neurons under control conditions, treated with roscovitine (RV) (15 μM), Aβ (5 μM) or Aβ + RV for 20 h. **(h)** Blots were quantified by densitometry and normalized against α-tubulin levels. The mean values ± SD of three independent experiments are shown (p < 0,05; p < 0,05). **(i)** Glutamate measurement in extracellular medium of neurons under different treatments (as in **d**). Mean values ± SD of three independent experiments are shown (p < 0,05).

We checked weather the glutamate increase caused by Aβ or proTAME led to a change in the Ca^2+^ level in neurons. Cytochemical analysis using Fluo4 showed that there was a rise in the intracellular Ca^2+^ level after 24 h of treatment with Aβ or proTAME. Such levels were similar to those of cells treated directly with glutamate (500 μM), which was used as a positive control. When glutaminase was inhibited in neurons that had been treated with proTAME, the increase in Ca^2+^ levels was abolished (Fig. 4a,b).

Next we analyzed the effect of Aβ or glutamate on the protein levels of cdk5, a kinase of cdh1 (Maestre *et al*.^10^), and of its Ca^2+^ dependent activators, p25 and p35. We observed that cdk5 and p25 increase in neurons upon both treatments after 24 h (Fig. 4c–f). To test the involvement of cdk5 in the lowering effect of Aβ on cdh1 levels, we used roscovitine, a well-known cdk inhibitor. We found that inhibition of cdk5 prevents the effect of Aβ on cdh1. Moreover, the Aβ**-**induced accumulation of glutaminase was partially prevented by roscovitine (Fig. 4g,h). This was accompanied by a slight attenuation of an Aβ-induced glutamate increase in the extracellular medium of neurons (Fig. 4i).

**Figure 5:**
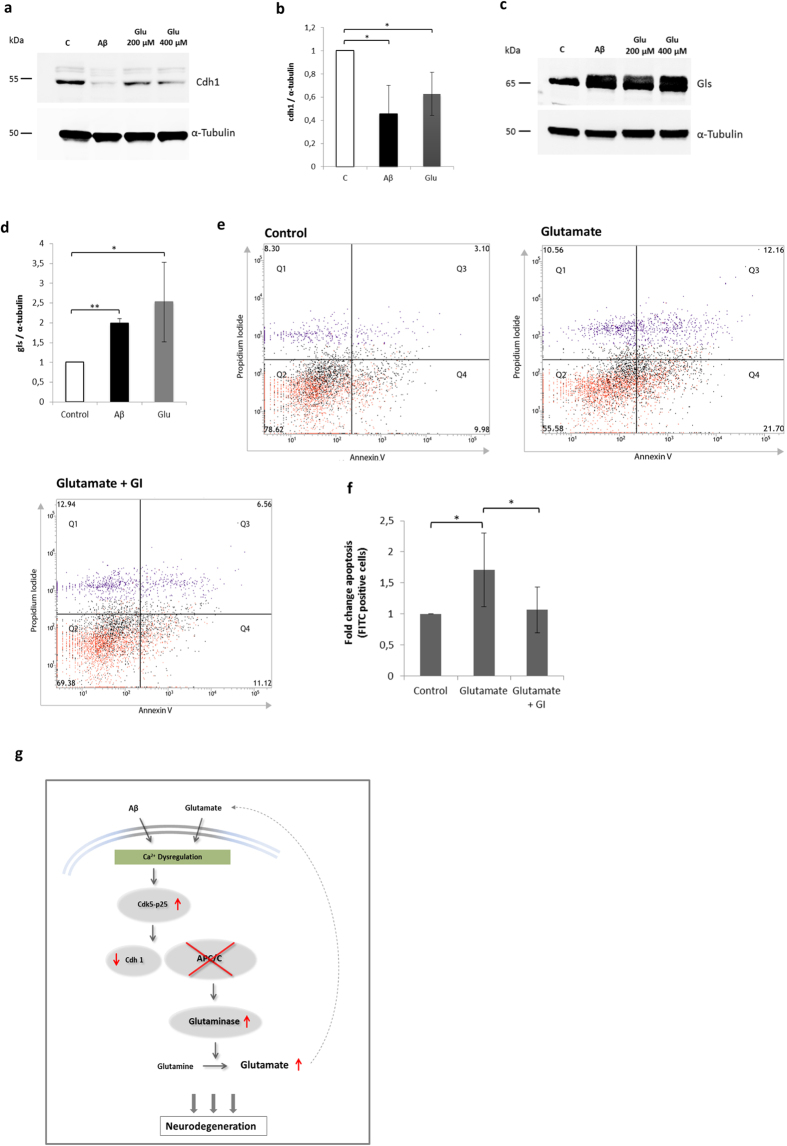
Glutamate decreases cdh1 and leads to glutaminase accumulation and apoptosis. **(a–d)** Representative Western blot images of cdh1 and glutaminase (gls) under control conditions, with Aβ or glutamate (200 μM or 500 μM) (glu) treatment. Blots were quantified by densitometry and normalized against α-tubulin levels. The mean values ± SD of cdh1 (p < 0,05; p < 0,05) and gls (p < 0,01; p < 0,05) of three independent experiments of controls, Aβ and glutamate (500 μM) are shown. **(e)** Representative assays of flow cytometry analysis for apoptosis of neurons under control conditions, treated with glutamate (500 μM) or glutamate + GI. **(f)** Mean FITC values ± SD are shown from data of four independent experiments (p_[C; Glut]_ < 0,01; p_[Glut; Glut+GI]_ < 0,01). **(g)** Graphical representation of the Aβ- and glutamate-induced alterations. Ca^2+^ dysregulation cause cdk5-p25 stabilisation and this leads to cdh1 decrease. Glutaminase accumulates and generates an increase in extracellular glutamate levels, which might induce a positive feedback loop of further APC/C-Cdh1 deactivation.

Since Ca^2+^ dysregulation might contribute to the Aβ-induced cdh1 decrease, we tested the effect of glutamate on cdh1 protein levels. We observed that glutamate treatment (500 μM) for 24 h caused a decrease in the cdh1 protein level, similar to that of Aβ (Fig. 5a,b). In the same samples we observed an accumulation of glutaminase (Fig. 5c,d). Then we checked whether the inhibition of glutaminase protects against apoptosis, induced by an excitotoxic stimulus of glutamate. Using flow cytometry we showed that glutamate-induced apoptosis was lowered when glutaminase was inhibited (Fig. 5e,f). Results were summarized in a mechanistic diagram (Fig. 5g).

### *In vivo* injection of Aβ or glutamate causes nuclear export of cdh1 in hippocampal neurons

**Figure 6:**
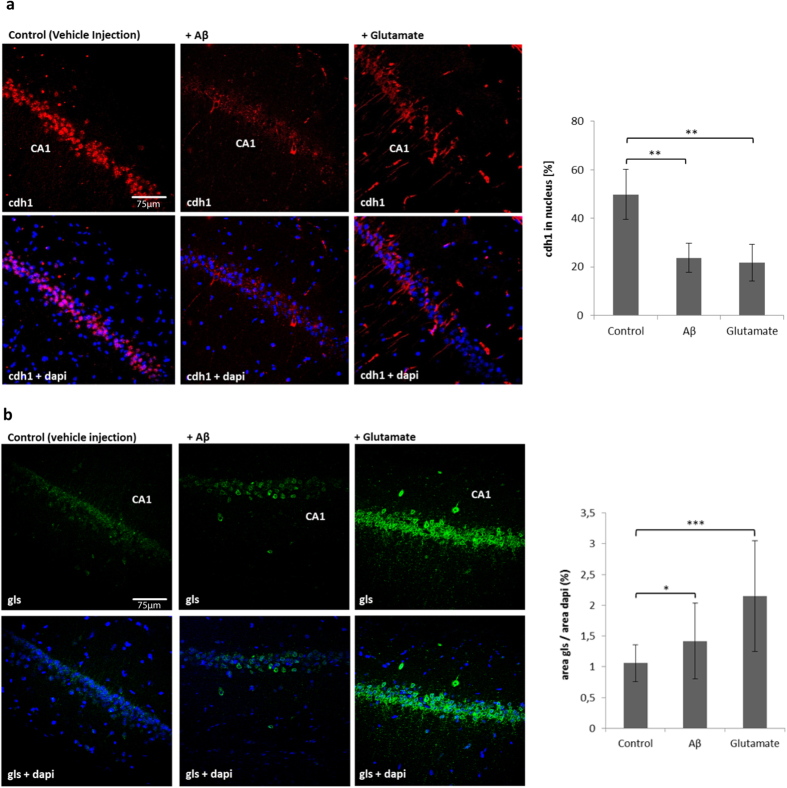
Cdh1 and glutaminase in hippocampal CA1 brain slices (40 μm) after microinjection of Aβ or glutamate. **(a)** Representative images of cdh1 and nucleus (dapi) stained hippocampal slices show a nuclear export of cdh1 when treated with Aβ or glutamate compared to vehicle injection. Results are shown as mean ± SD of images of three independent experiments (3 animals were used for each treatment (C, Aβ and glutamate); within each group 3 series (triplicates) were analysed); percentage of nuclear cdh1 (p_[C; Aβ]_ < 0,01; p_[C;Glut]_ < 0,01) is shown. **(b)** Representative images of glutaminase (gls) and nucleus (dapi) stained hippocampal slices show that glutaminase levels increase upon treatment with Aβ or glutamate. Results are shown as mean ± SD of three independent experiments (3 animals were used for each treatment (C, Aβ and glutamate); within each group 2 series (duplicates) were analysed) glutaminase area normalized to dapi area (p_[C; Aβ]_ < 0,05; p_[C; Glut]_ < 0,001).

To test the effect of Aβ or glutamate on cdh1 *in vivo* we used microinjection in the brain of Wistar rats. We injected 10 μl volume of vehicle solution, Aβ (5 μM) or glutamate (1 mM) in the CA1 region of the hippocampus according to stereotaxic guidelines and two days later the animals were sacrificed. Immunohistochemical analysis showed that cdh1 is located in the nucleus and in the cytoplasm in control conditions (vehicle injection) in CA1 neurons. Injection of Aβ or glutamate causes a translocation of cdh1 from the nucleus to cytoplasm, which resulted in a 25% decrease of nuclear cdh1 (Fig. 6a). Both treatments caused an increase in glutaminase in neurons in the pyramidal CA1 layer (Fig. 6b).

### APP/PS1 mice: The cdh1 protein level is lower and glutaminase is increased in the transgenic AD animal model

To further test the role of APC/C-Cdh1 in AD pathophysiology we used the amyloid precursor protein/presenilin 1 (APP/PS1) mouse model of AD. APP/PS1 mice are subjected to chronic exposure to Aβ. We compared the protein levels of cdh1 and glutaminase in wild type (WT) and APP/PS1 mice at the age of 9 months (n = 6 WT, n = 6 APP/PS1).

**Figure 7:**
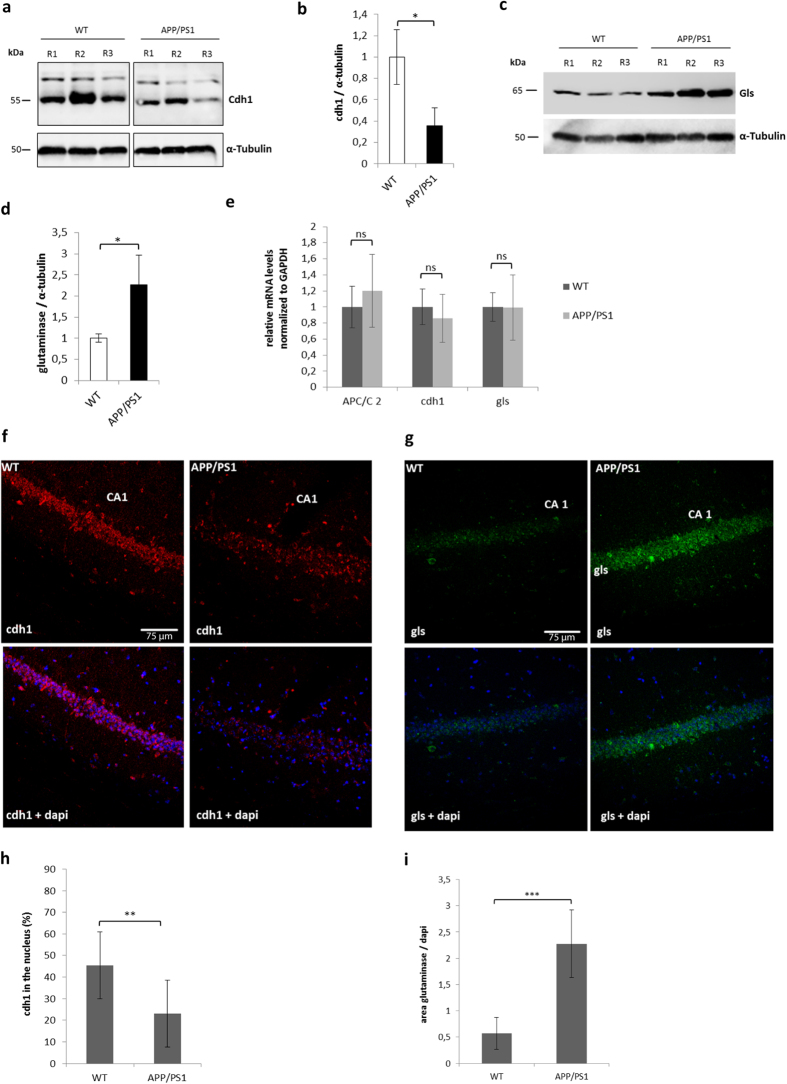
Cdh1 and glutaminase are altered in APP/PS1 mice. **(a–d)** Western blot analysis of cdh1 and glutaminase (gls) protein levels measured in homogenates of mouse cerebral cortex of 3 wild type (WT) and 3 transgenic APP/PS1 mice [images of cdh1 bands are from the same blot]. Cdh1 and gls were quantified by densitometry and normalized against α-tubulin levels, mean ± SD of three independent samples are shown in the histogram. **(e)** Mean values ± SD of mRNA levels of APC/C2, cdh1 and glutaminase normalized to GAPDH in cortex homogenates of WT and APP/PS1 mice are shown. **(f–g)** Representative images of cdh1 and nucleus (dapi) stained hippocampal slices of each 3 WT and 3 APP/PS1 mice (3 series per animal were analysed). Results are show as mean ± SD of percentage of nuclear cdh1 (p < 0,01). **(h–i)** Representative images of gls and nucleus (dapi) stained hippocampal slices of each 3 WT and APP/PS1 animals (2 series per animal were analysed). Results are shown as mean ± SD of glutaminase area normalized to dapi area (p < 0,001).

Using Western blot analysis, we observed significantly lower levels of cdh1 in cortex homogenates of APP/PS1 compared to WT mice. Analysis of glutaminase in the same samples showed higher levels in the APP/PS1 than in the WT mice (Fig. 7a–d). We also measured mRNA levels of cdh1 and APC/C2 and glutaminase in cortex homogenates in each 3 WT and 3 APP/PS1 mice using qPCR. We did not detect any significant difference in their expression level (Fig. 7e).

Perfusion of 3 WT and 3 APP/PS1 animals was carried out for immunohistochemical analysis. We observed less cdh1 in the nuclei of APP/PS1 animals compared to WT in the hippocampal CA1 region. Measurement of glutaminase showed significantly increased levels in APP/PS1 compared to WT animals (Fig. 7f–i).

## Discussion

APC/C-Cdh1 plays an essential role in neurons, and dysregulation of this ubiquitin ligase has been associated with neurodegeneration^3^. However, the role of APC/C-Cdh1 in the pathophysiology of AD remains unclear. In this work we show that soluble oligomeric Aβ decreases cdh1 protein levels *in vitro* and *in vivo*. Here we report for the first time, results that indicate a direct implication of APC/C-Cdh1 in AD. Moreover, we found evidence that uncontrolled accumulation of glutaminase, an APC/C-Cdh1 degradation target, contributes to the generation of an excitotoxic environment and neuronal apoptosis.

We found evidence that suggests that cdk5-p25 is involved in the Aβ-induced degradation of cdh1 in neurons. It has been reported that Aβ causes a dysregulation in Ca^2+^ homeostasis which leads to the stabilization of p25, the activator of cdk5^19^. Furthermore, cdk5-p25 accumulates in the brains of AD patients and promotes neurodegeneration^20^. Maestre *et al*.^10^ showed that cdk5-p25 phosphorylates cdh1 through a Ca^2+^ mediated mechanism when glutamate receptors were overactivated. Phosphorylated cdh1 is translocated from the nucleus to the cytoplasm^11^, where it is then degraded^3^. Here we showed that Aβ treatment in neurons in culture causes an increase in intracellular Ca^2+^ levels and cdk5-p25, which seems to be involved in the decrease in cdh1. However, further experimental work will be needed to confirm whether the Aβ induced cdh1 decrease is due to cdk5. Taking our results and the previous findings together, there is evidence for an Aβ-induced Ca^2+^ – cdk5-p25 – cdh1 signaling alteration in AD.

A decrease in APC/C-Cdh1 activity causes a subsequent accumulation of the degradation targets of the ubiquitin ligase. Undesired increases of some of those target proteins have been related to neurodegeneration^10^,^21^. Here, we measured protein levels of cyclin B1 and glutaminase. Whilst cyclin B1, a well-described target of the ubiquitin ligase was used as an indicator of APC/C-Cdh1 activity^22^, we focused on the significance of glutaminase accumulation.

Alterations in glutaminase levels have been associated with AD. It was found that in brains of AD patients, glutamate and glutaminase stained neurons contained neurofibrillary tangles. Moreover, glutamate and glutaminase immunoreactive pyramidal neurons in the hippocampal CA1 were decreased. The remaining neurons showed irregular shortened and disorganized dendritic fields^23^. Burbaeva *et al*.^24^ reported increased levels of glutaminase in the prefrontal cortex of AD patients. In the present work we have found that APC/C inhibition by proTAME or Aβ treatment leads to glutaminase accumulation in neurons. We also showed that depletion of cdh1 using siRNA induces an increase in glutaminase, supporting the idea that cdh1 depletion may be the primary mechanism of glutaminase elevations caused by Aβ or the APC/C inactivation by proTAME treatment.

Moreover, we found that the inhibition of APC/C generates increased glutamate levels in the extracellular medium. High levels of glutamate have been observed in the cerebrospinal fluid of AD patients compared to healthy individuals of a similar age^15^,^16^,^25^,^26^. Currently, glutamatergic systems are one of the main therapeutic targets in AD treatment^27^ and Aβ synaptic toxicity can be partially ameliorated by the N-methyl-D-aspartate receptor (NMDAR) antagonist memantine^28^,^29^. In several studies the molecular mechanism of excitotoxicity was attributed to failure in the glutamate recycling system. Aβ decreases the glutamate transporter GLT1 and therefore glutamate cannot be properly taken up by astrocytes and thus the neurotransmitter remains in the synaptic cleft^30^,^31^. We postulate here that excitotoxicity in AD does not only result from perturbations in the glutamate reuptake system, but also from aberrantly increased glutamate generation by glutaminase. We showed that Aβ increased glutamate levels in the extracellular medium of neurons and that this may be mediated by inactivation of APC/C. Direct inhibition of APC/C was sufficient to increase the glutamate concentration to similar levels as those found after Aβ treatment. This was mediated by glutaminase. Removal of glutamine from the culture medium completely abolished the Aβ- or proTAME-induced glutamate increase. This further indicates that it is the enzymatic reaction catalyzed by glutaminase that gives rise to the aberrant glutamate level. Moreover, we found that Aβ- or proTAME treatment induced neuronal apoptosis, which was ameliorated by the inhibition of glutaminase. This indicates that increased glutaminase levels, and resultant glutamate levels, induced excitotoxicity after the treatments with Aβ or proTAME.

Interestingly, Maestre *et al*.^10^ reported that an excitotoxic glutamate stimulus causes APC/C-Cdh1 inactivation. We have confirmed here that glutamate reduces cdh1 protein levels and have shown that it also leads to glutaminase accumulation. Moreover, glutamate-induced neuronal apoptosis was ameliorated when glutaminase was inhibited. It has been reported that an excitotoxic glutamate insult causes sustained NMDA receptor activation underlying a positive feedback loop^21^,^32^. Our findings suggest that glutamate-induced glutaminase accumulation may contribute to a positive feedback loop of glutamate generation resulting in excitotoxicity, and thereby maintaining the down-regulation of APC/C activity. This would lead to the accumulation of APC/C-Cdh1 targets, of which some were related to neurodegenerative processes. An aberrant cyclin B1 increase in neurons causes an ectopic cell cycle re-entry and high levels of 6-phosphofructo-2-kinase/fructose-2,6-bisphosphatase (pfkfb3) induce oxidative stress^10^,^33^. Moreover, gene profiling has shown that excitotoxicity favors neuronal pathways that induce cell cycle re-activation and oxidative stress and that these events, together, accelerate neurodegeneration^34^. We suggest these events, which all occur in AD, are linked through deficient APC/C activity.

We then turned to *in vivo* studies using acute (injections) as well as chronic (APP/PS1 transgenic mice) insults. Microinjection of Aβ_1-42_ oligomers in the CA1 field of rat hippocampus caused nuclear export of cdh1 and increased glutaminase levels. It has been reported in a study using similar experimental setups, that intrahippocampal microinjection of Aβ_1-42_ oligomers impaired spatial working memory in rats^35^. We then analyzed cdh1 protein levels in the APP/PS1 mouse model of AD, in which neurons are chronically exposed to Aβ. We found lower levels of cdh1 (and predominantly less cdh1 in the nucleus) than in WT mice. Glutaminase was increased in the same brain areas. Thus we confirmed our results in an *in vivo* model of AD.

Similar to the effects of Aβ injection, glutamate stimulus caused nuclear export of cdh1 in the hippocampus, and glutaminase was also increased compared to controls. These findings might also be relevant for other neurodegenerative diseases. It has been reported that global ischemic injury induces downregulation of APC/C-Cdh1 in the CA1 field of the hippocampus in rat brain, and this was related to neuronal apoptosis^36^. Ischemic injury causes an increase in the extracellular glutamate concentration leading to over-stimulation of the NMDA receptors in extrasynaptic sites. Proteins involved in this excitotoxic cascade are, among others, PTEN, calpain, cdk5, p25. These events cause delayed and progressive neuronal damage and neuronal death (damage appears three days post-ictus and continues progressively for months)^37^. Our observation of the effect of glutamate on cdh1 suggests that cdh1 down-regulation in ischemia observed by Zhang *et al*., might have been induced by glutamate excitotoxicity. Moreover, APC/C-Cdh1 down-regulation and subsequent glutaminase accumulation may contribute to the generation of excitotoxic environments in ischemia.

In several knock-out models of cdh1, impaired long-term potentiation (LTP) and long-term depression (LTD) has been described^38^,^39^,^40^. Defects in synaptic plasticity have been related to neurodegenerative diseases. Interestingly, in the APP/PS1 mouse model, alterations in LTP have been described^41^. Therefore, we hypothesize the cdh1 decrease that we observed in APP/PS1 mice, might contribute to the defects of LTP in these mice.

Recent studies, using functional MRI, showed an increase in neural network activities in prodromal AD subjects relative to baseline. Therefore, a new concept of Aβ-mediated slow excitotoxicity in early stages of the disease was introduced. Growing evidence supports the view that this may be critically involved in neurodegeneration in AD^42^. We suggest that our findings of the Aβ-Ca^2+^-cdk5-p25-cdh1-glutaminase-glutamate pathway might be one of the molecular links between the pathogenic factors Aβ and slow excitotoxicity. Thus we suggest that this pathway may be an interesting target in AD research and treatment.

## Materials and Methods

### Preparation of Amyloid beta peptide

We added an alkaline solution, 50 μl of NH_4_OH (1% in 1x PBS) or 20 μl DMSO, to 1 mg of Aβ_(1-42)_ (amyloid-beta 1-42, 20276, AnaSpec, Fremont, USA), which dissolves immediately. Subsequently, a stock solution of 100 μM Aβ was prepared in 1x PBS. We incubated the solution at 4 °C for 24 h to allow the formation of oligomers. It was then used immediately or stored at −20 °C and used within 2 months after preparation^43^,^44^,^45^.

### Cell culture, transfections and treatments

Primary cultures of cortical neurons were prepared from brains of Wistar rat fetus at 14 days of gestation. Cortices were dissected mechanically, dispersed in serum free culture medium and the tissue was filtered through a nylon net (90 μm pores). Neurons were seeded on polylysine-covered T_25 _culture flasks (surface area 25 cm^2^) for Western blot, glutamate and flow cytometry analysis. For fluorescent microscopy neurons were seeded on polylysine-covered chambered coverglass slides (surface area 0,7 cm^2^). They were cultured in DMEM supplemented with fetal bovine serum (10% v/v) and antibiotics penicillin and streptomycin mix (1%), incubated at 37 °C in a 5% CO_2_-containing atmosphere. At 72 h after plating, cytosine-arabinosid (10 mM) was added to the culture medium, and one day later it was replaced by culture medium containing the half amount of cytosine-arabinosid. The following day, the medium was removed and replaced by standard culture medium. After 6 days, we obtained a 95% pure neuron culture, which was used for treatments. The culture medium was replaced by fresh culture medium by the time of treatments, which were performed at the following concentrations if not indicated otherwise: Aβ oligomers (5 μM), glutamate (500 μM) (G-1251, Sigma, St. Louis, USA), proTAME (12 μM) (I-440, BostonBiochem, Cambridge MA, USA), compound 968 (100 μM) (352010, Calbiochem, Nottingham, UK), roscovitine (15 μM) (R 7772, Sigma, Missouri, USA); cycloheximide (3,5 μM) (C7698 Sigma, Missouri, USA), MG132 (10 μM) (M7449, Sigma, Missouri, USA); the incubation times are indicated in figure legends. Neurons were analysed after 7 days as described below. For cdh1 silencing experiments we used Accell SMARTpool Fzr1 siRNA (E-100432-00-0050, Dharmacon), and Accell non-targeting siRNA (D-001910-01-20, Dharmacon): 1 μM siRNA per ml culture medium with reduced serum to 1% for 96 h. Then the cells were collected and used for treatment.

### Transgenic Animals

APPswe, PSEN1dE9-85Dbo/J transgenic mice and wild type mice from the same colony aged nine months were used in this study. Mice were maintained individually under a 12:12-h dark-light cycle at 23 ± 1 °C and 60% relative humidity, and were provided with a standard chow diet (PANLAB S.L.) and water ad libitum.

### Preparation of tissue for Western Blot

The animals were anesthetized with inhalatory anesthesia (SEVOrane^®^) and then sacrificed. Cortices were isolated, freeze-clamped and stored at −80 °C. For Western blot analysis, the tissues were weighed and the appropriate amount of 1x lysis buffer (Tris: 76.5 mM; pH: 6.8; SDS: 2%; Glycerol: 10%; supplemented with sodium ortovanadate (2 mM) and protease inhibitor (Sigma-Aldrich)) was added (1 ml 1x lysis buffer/100 mg brain tissue) and the tissues were homogenized using mechanical shear with a Potter-glass-Teflon homogenizer (Rw20 DZM Homogenizer, Janke & Kunkel), at 2000 rpm in ice. For immunohistological analysis, animals were transcardially perfused as described below.

### Intrahippocampal infusion

A group of nine female Wistar rats were treated with atropine methyl nitrate (0.4 mg/kg, i.p.), anesthetized with a mixture of Ketamine (60 mg/kg i.p.) (Imalgen, 0.05 g/ml; Rhone Mérieux, Lyon, France) and Xylazine (10 mg/kg i.p.) (Xilagesic, 20 mg/ml; Lab. Calier, Barcelona, Spain), and mounted in a Kopf stereotaxic apparatus (Kopf Instruments, Tujunga, USA). In order to minimize animals’ suffering, subcutaneous lidocaine was injected in the surgery area. The scalp was incised and retracted, and the head position was adjusted to place bregma and lambda references in the same horizontal plane. Small trephine holes (1 mm in diameter) were drilled in the skull unilaterally.

Intrahippocampal infusions were made using a stainless steel cannula system (Plastics One, Roanoke, VA) consisting of an outer guide tube (24 gauge) and an inner infusion tube (31 gauge). The cannulas were stereotaxically placed 4.36 mm posterior to bregma, 1.4 mm lateral from the mid-sagittal line, and implanted 3.4 mm below the outer surface of the skull into the left-side CA1 hippocampal region, according to the atlas of Paxinos and Watson 1998. The infusion cannula was connected to a 10 μl Hamilton microsyringe by a polyethylene tube. A total volume of 10 μl of infusion solutions (1 mM glutamate in 1x PBS; 5 μM Aβ dissolved in 0.5% DMSO in 1x PBS) were injected into hippocampus at 0.5 μl/min controlled by a syringe pump. Control rats received vehicle solution (same volume and same rate).

### Fixation and tissue preparation

After 48 h of treatment, animals were anaesthetised with a lethal dose of sodium pentobarbital (100 mg/kg 20%) (Dolethal Vetoquinol Madrid, Spain) and transcardially perfused with heparinized saline (0.1%, pH 7) followed by paraformaldehyde (4%) in phosphate buffer (PBS) (0.1 M, pH 7.4). Brains were post-fixed for 24 h in paraformaldehyde (4%) in PBS at 4 °C and maintained for 2 days in sucrose (30%) at 4 °C for cryoprotection. Coronal sections of 40 μm were obtained by freezing microtomy (Leica) and stored in phosphate buffer (0.1 M, pH 7.4) at 4 °C.

### Immunohistochemical labelling

Free floating sections were washed in Tris buffered saline with Triton solution (0.1%) (TBS-Tx), 3 times at room temperature (RT). Sections were incubated for 3 h at RT in a pre-incubation solution of 2% bovine serum (BSA; A7906, Sigma) in TBS-Tx. After the pre-incubation, the tissue was incubated, 2 days at 4 °C, in a primary solution containing BSA (2%) in TBS-Tx with the primary antibodies: rabbit anti-Glutaminase (Proteintech; 1:200, Chicago, USA), mouse anti-Cdh1 (Abcam; 1:100, Cambridge, UK) or rabbit anti-Cdh1 (NBP2, Novusbio; 1:250, Cambridge, UK). Afterwards, sections were rinsed 2 times in TBS-Tx and 2 times in HEPES (10 mM) in physiological saline solution and then incubated for 3 h at RT in a solution of 2% BSA in HEPES (10 mM) with the secondary antibody: Alexa Fluor 488 anti-rabbit (CellSignaling Tech; 1:1000, Danvers, USA) and Alexa Fluor 647 anti-mouse (CellSignaling Tech; 1:1000). Finally, sections were rinsed in HEPES (10 mM), mounted onto pig skin gelatin-coated slides (0.5%) and covered with a drop of VECTASHIELD Mounting Medium with DAPI (H1200, Vector, Burlingame, USA) for nuclear staining and a drop of fluorescence mounting medium (S3023, DAKO, Barcelona, Spain) was added.

### Immunocytochemical labelling

For calcium staining in living cells, fluo 4 (F-14201, Invitrogen, Oregon, USA) was used. Five μl fluo 4 (50 μg fluo4 in 50 μl DMSO) were added to 1 ml medium of neurons and they were incubated for 45 min at 37 °C. Afterwards they were washed in 1x PBS and further incubated for 30 min. Nuclei were stained with Hoechst (H3570, Invitrogen). For cdh1 analysis in neurons, they were fixed in formaldehyde (4% in 1x PBS) and stained using anti-cdh1 (1:100, NBP2-15840, Novusbio) and Map2 (1:100, M9942, Sigma). Fluorescent secondary antibodies were used as above. Nuclei were stained with Hoechst (H3570, Invitrogen).

### Image acquisition and data analysis

Immunocytochemical and immunohistochemical preparations were visualised with a confocal laser-scanning microscope (Leica TCS SP2 scanning multiphoton and confocal unit with an inverted DM1RB microscope; Ar-He-Ne). Images containing hippocampal CA1 regions were analysed with *ImageJ*. To determine the nuclear and cytoplasmic localization of cdh1 we used the “intensity ratio nuclear cytoplasm” plugin for *ImageJ* with threshold setting *Huang*.

To compare protein levels we used area proportions of the specific antibody signal normalized to nuclear stained area (dapi). Ca^2+^ quantifications were performed by intensity level analysis of Fluo4 normalized to nuclear staining (hoechst) using *ImageJ*; all images were taken with the same exposure settings.

### Western blot analysis

Neurons from cell culture were collected and lysis buffer was added. Tissue homogenates were prepared as described above. Samples were boiled for 5 min and stored for analysis at −80 °C. Protein concentrations were determined using Lowry protein assay. The same amount of total protein in the samples (20 μg) were subjected to SDS polyacrylamide gel electrophoresis (in running buffer: 25 mM Tris, 190 mM glycine, 0,1% (w/v) SDS) and blotted onto a nitrocellulose membrane in a wet-transfer system (in transfer’s buffer: Tris 25 mM, 192 mM glycine, 20% (v/v) methanol).

The membranes were blocked in 5% low-fat milk or 5% BSA (w/v) in 1x TBS-Tween for 1 h at room temperature and were incubated overnight at 4 °C with primary antibodies in blocking solution: cdh1 (DCS-266, 1:500, Novus Biologicals), glutaminase 1 (12855-1-AP, Proteintech, 1:1000), cdk5 (2506, Cell Signaling Technology, 1:1000), p35/25 (2680, 1:1000, Cell Signalling Technology), cdc20 (4823, Cell Signalling Technology, 1:1000), α-Tubulin (sc-8035, Santa Cruz Biotechnology, 1:8000, Heidelberg, Germany). Membranes were incubated with corresponding secondary antibodies and signal detection was performed using “Luminata Classico Western HRP Substrate” (WBLUC0500, Millipore Corporation, Billerica, USA). Western blot images were developed with a biomolecular imager (ImageQuant™ LAS 4000, GE Healthcare Bio-Sciences). Densitometry of Western blot images was accomplished using *ImageGauge4.0.*

### Glutamate Measurement

To measure L-glutamate we followed the protocol of Bergmeyer, H.U. & Bernt, E. “UV-Assay with Glutamate Dehydrogenase and NAD”^46^, a photometric assay, based on the enzymatic reaction of glutamate dehydrogenase (GlDH, G2626, Sigma, St. Louis, USA), adapted for a 96-well plate reader. Samples of cell-free supernatants of cultured neurons were deproteinized using perchloric acid (6%). The pH was adjusted using tripotassium phosphate solution. The reaction of GlDH leads to an increase of NADH, which is measured by the absorbance change at 340 nm and is proportional to the amount of L-glutamate. The concentration of glutamate in the supernatant was normalized to 100 μg protein of corresponding neuron cell lysates.

### Flow Cytometry

For apoptosis assays, neurons were analysed with a BD FACSVerse™ flow cytometer using the annexin V-FITC Apoptosis Detection Kit (ANEXVKF-100T, Immunostep, Salamanca, Spain).

Cells were detached from culture plates with Trypsin (2 min, 37 °C), collected and added to floating cells from the supernatants. Neurons were washed twice in 1x PBS and re-suspended in 100 μl Annexin Buffer. Five μl of both, Annexin and Propidium Iodide (PI) were added to the cells for 15 min at RT. Neurons were further diluted in Annexin Buffer and analysed by flow cytometry. The measurements were previously calibrated using neurons with either Annexin or PI staining and unstained cells. Flow cytometry results were analysed with BD FACSuite software. Results are shown as 2D dot plots, with Annexin V**-**FITC on the x-axis and PI on the y-axis. The bivariate staining allows discrimination of the following groups: intact cells in Q2 (Annexin negative, PI negative) in the square left/down-sided, early apoptosis Q3 (Annexin positive PI, negative) in the square right/down-sided, Q4 late apoptotic (Annexin positive, PI positive) in the square right/up-sided; or necrotic cells Q1 (Annexin negative, PI positive) in the square left/up-sided. Histograms show the mean ± SD of at least 3 independent experiments of each treatment condition.

### Determination of mRNA expression by quantitative rtPCR

Total RNA was isolated from tissues or cells using the TRIzol Reagent (Life technologies, 15596-026) according to manufacturer’s instructions. RNA concentration was determined using a NanoDrop spectrophotometer. One μg of purified RNA was reverse transcribed using a ‘High-Capacity cDNA Reverse Transcription Kit’ (Thermo Fisher, 4368814) according to the manufacturer’s instructions. Gene-specific primer pairs were designed using NCBI/Primer-BLAST:

cdh1 (Mus musculus) FW: 5′-GGACCAGGACTATGAGCGAA-3′,

cdh1 (Mus musculus) RV: 5′-GGGTTCTCCGCATCTCTGAA -3′;

APC/C 2 (Mus musculus) FW: 5′-ACCGTATCTATGCCACCCTAC-3′,

APC/C 2 (Mus musculus) RV: 5′-GACACAAGAAGTTGCTGCCT-3′;

gls (Mus musculus) FW: 5′GATGTGTTGGTCTCCTCCTCT-3′,

gls (Mus musculus) RV: 5′-GGTTTATCACCGACTTCACCC-3′;

GAPDH (Mus musculus) FW: 5′- TGCTGAGTATGTCGTGGAGT-3′,

GAPDH (Mus musculus) RV: 5′-AGATGATGACCCGTTTGGCT-3′;

cdh1 (Rattus norvegicus) FW: 5′- TCGTATCGTGTCCTCTACCTG-3′,

cdh1 (Rattus norvegicus) RV: 5′- AACCGAAGGGTCTCATCTCC-3′;

APC/C2 (Rattus norvegicus) FW: 5′- GAGAGAGTGGTTGGTTGGCT-3′,

APC/C2 (Rattus norvegicus) RV: 5′-GGCTGGCATAGATTCGGTAGA-3′;

gls (Rattus norvegicus) FW: 5′ ATTACGACTCCAGAACAGCCC,

gls (Rattus norvegicus) RV: 5′-GCTTCC AGCAAAAACTTCACAAC-3′;

GAPDH (Rattus norvegicus) FW: 5′ TGATGGG TGTGAACCACGAG-3′,

GAPDH (Rattus norvegicus) RV: 5′-TCATGAGCCCTTCC ACGATG′-3.

The primers were obtained from *Life Techonologies*. For the reaction, Maxima SYBR Green/ROX qPCR Master Mix (2X) (Thermo Scientific, K0223) was used and each sample was analysed in triplicates. Quantitative PCR’s were performed using the detection system 7900HT Fast Real-Time PCR System (Applied Biosystems, Foster City, CA).

The relative standard curve method was used to evaluate the expression levels. Standard curves were prepared from dilutions of cDNA mix of the samples for each gene. The efficiency was estimated using a semi-log regression line plot of CT value vs. log of input nucleic acid. For all experimental samples, the relative target quantity was determined by interpolating the threshold cycle (Ct) values from the standard curve. GAPDH was used in all samples to normalize gene expression for sample-to-sample differences in RNA input and quality. The fold change of samples of the control group compared to treated groups was represented.

### Sample numbers and statistical analysis

The results are expressed as mean values of the data of at least three independent experiments; the error bars represent SD. Generally, experiments were performed as duplicates, within each independent experiment. Only when the sample number was high (in time course experiments), no replicates were carried out, however in this case, samples were analysed in duplicate by Western blot and glutamate measurements. All PCR reactions were carried out in triplicate. For immunohistochemical analysis, the sections of each brain were serially collected in three to five independent pools of samples (series). From these, at least two series were analyzed per animal (duplicate).

To calculate protein half-life we used the following method: It was assumed that protein degradation follows first-order decay kinetics. The measured protein intensity data was initially log-transformed, then a linear least-squares fit was used to determine the decay rate constant k, yielding a coefficient of determination (r-square). Moreover, we estimated the standard error of the slope. For assessing difference significance we used a Z-test. It is known that, under normality assumption of the fit errors, difference between slope estimates divided by the mean square root of their standard error follow a standard normal distribution. Finally, from the decay rate constant, the half-life was calculated (T(1/2) = ln(2)/k).

For other statistical analyses, we used one-tailed T-tests or Mann Whitney U-test analysis. P-values were ranked in the units of significance which were set as following: p > 0,05 not significant (ns), p < 0,05 significant (*), p < 0,01 very significant (**), p < 0,001 highly significant (***).

### Ethics statement

The methods in this manuscript were carried out in accordance with the approved guidelines. The use of animals and all experimental procedures were approved by “Comisión de Etica en la Investigación Experimental, Vicerectorado de Investigación” (University of Valencia) with the number: A1396426380485^46^.

## Additional Information

**How to cite this article**: Fuchsberger, T. *et al*. Aβ Induces Excitotoxicity Mediated by APC/C-Cdh1 Depletion That Can Be Prevented by Glutaminase Inhibition Promoting Neuronal Survival. *Sci. Rep.* **6**, 31158; doi: 10.1038/srep31158 (2016).

## Supplementary Material

Supplementary Information
