# Supplementary material for: Aβ Induces Excitotoxicity Mediated by APC/C-Cdh1 Depletion That Can Be Prevented by Glutaminase Inhibition Promoting Neuronal Survival
Source: Sci Rep. 2016 Aug 12;6:31158. doi: 10.1038/srep31158 (PMC4981891; doi:10.1038/srep31158)
Supplement: Supplementary Information [file srep31158-s1.doc]

**Aβ Induces Excitotoxicity Mediated by APC/C-Cdh1 Depletion That Can Be Prevented by Glutaminase Inhibition Promoting Neuronal Survival**

Fuchsberger T, Martínez-Bellver S, Giraldo E, Teruel-Martí V, Lloret A, Viña J

**a**

**Control**

**Glutaminase Inhibitor ‘compound 968’**


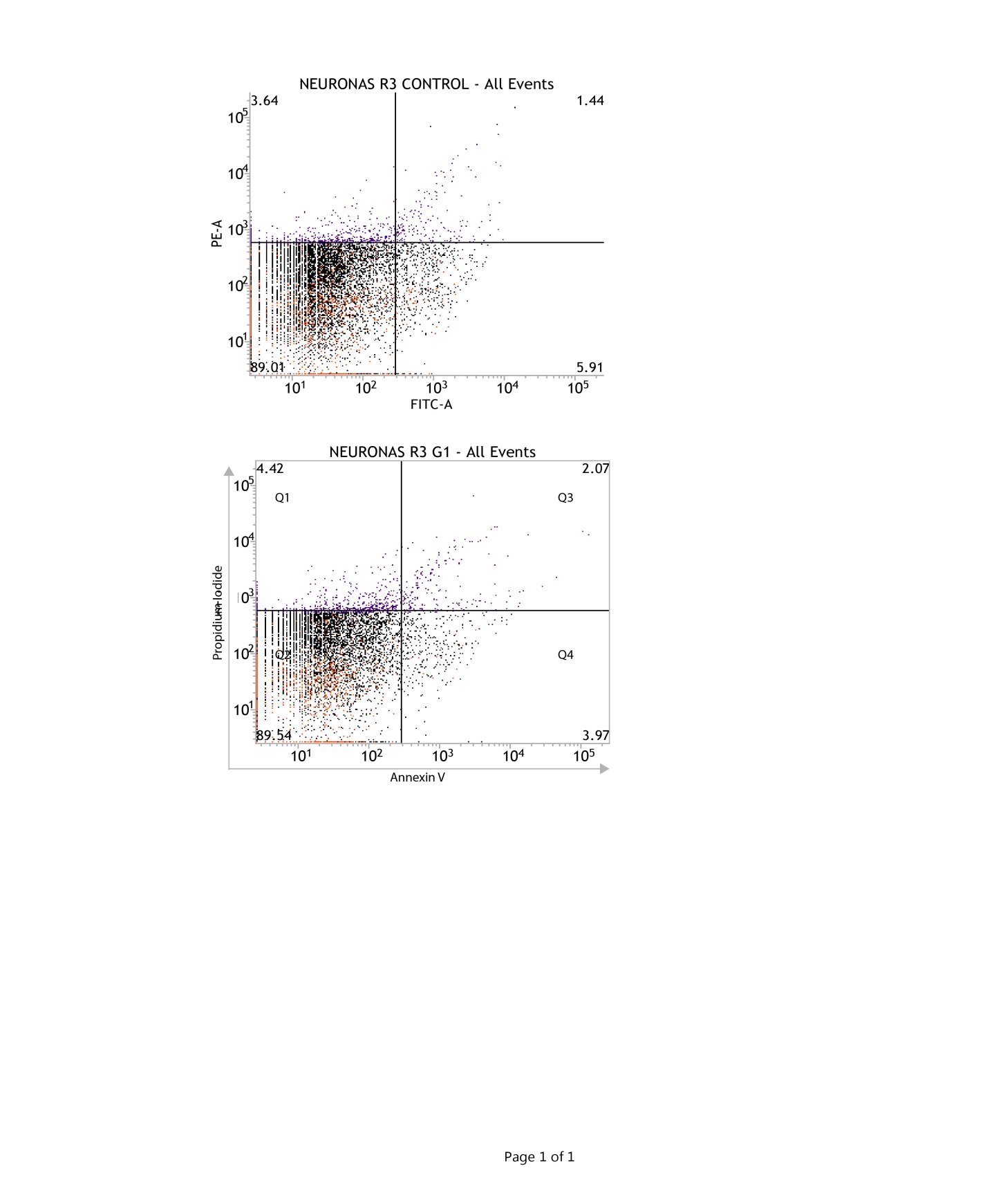

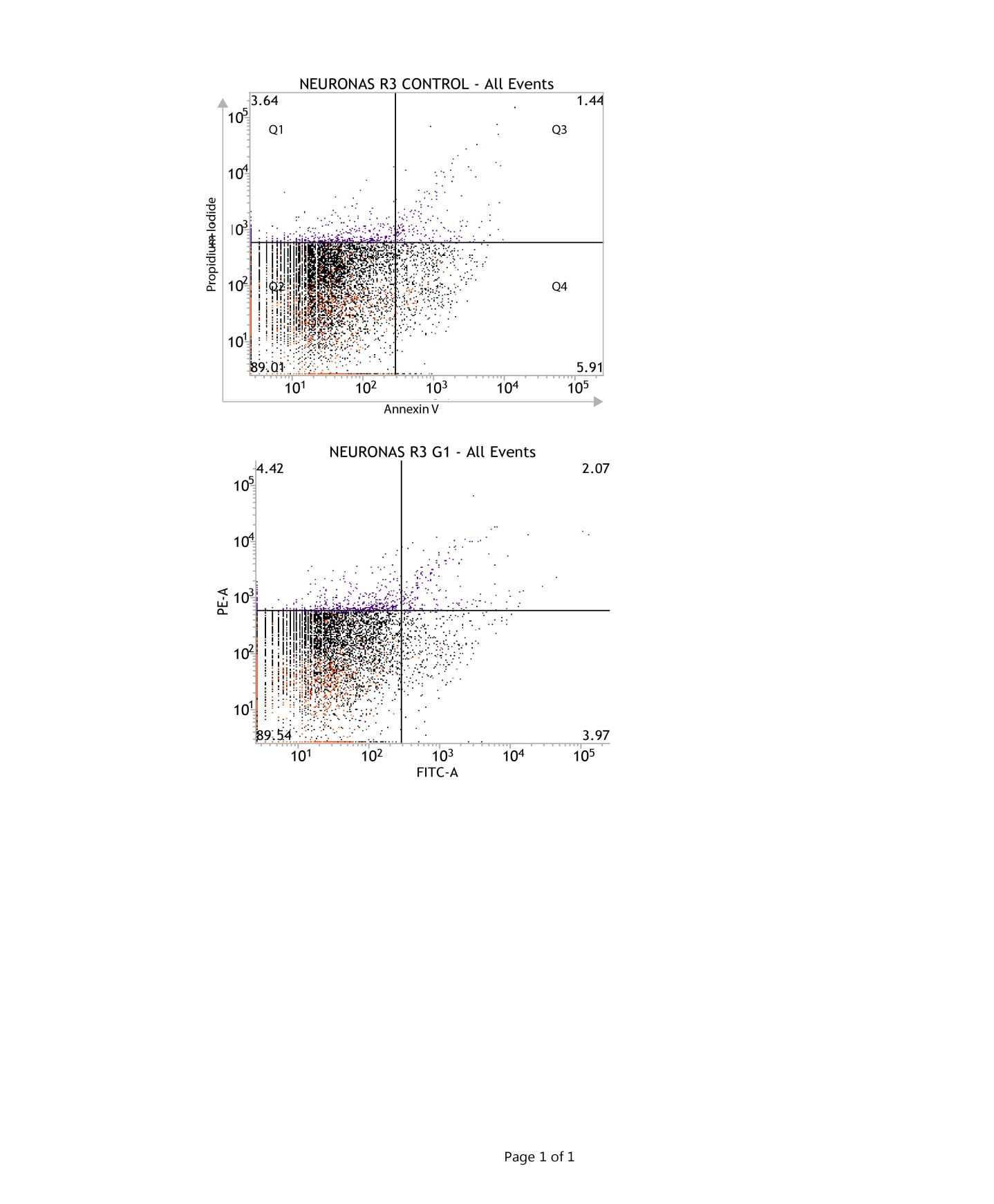


**b**


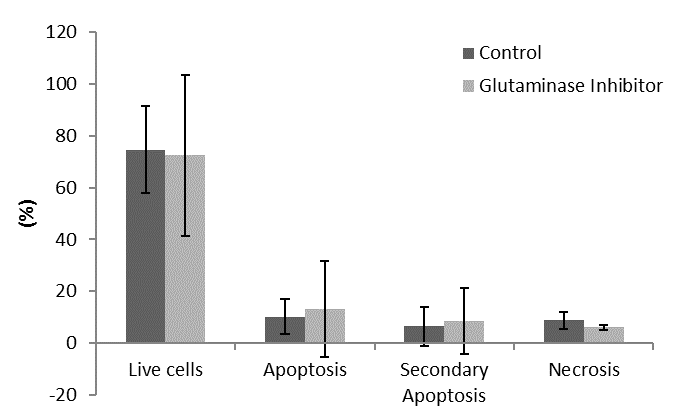


**Supplementary Figure 1 | (a)** Representative assay of flow cytometry analysis for apoptosis in neurons under control conditions, or treated with the glutaminase inhibitor ‘compound 968’ for 20 h. **(b)** Mean FITC values ± SD are shown from data of three independent experiments.

**
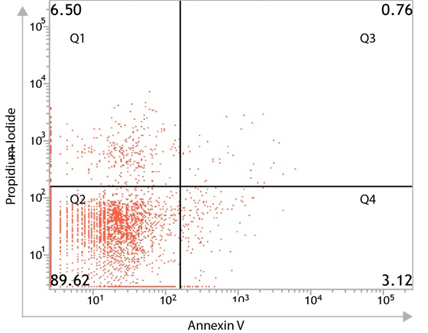

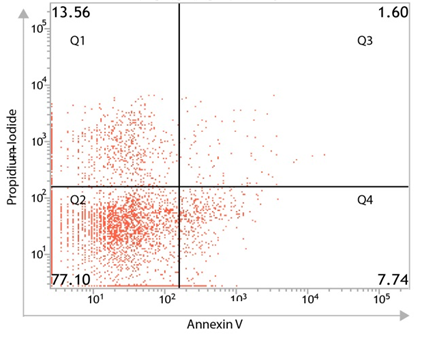

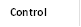
**

**pT 0 h**

**pT 4 h**

**pT 10 h**

**pT 16 h**

**
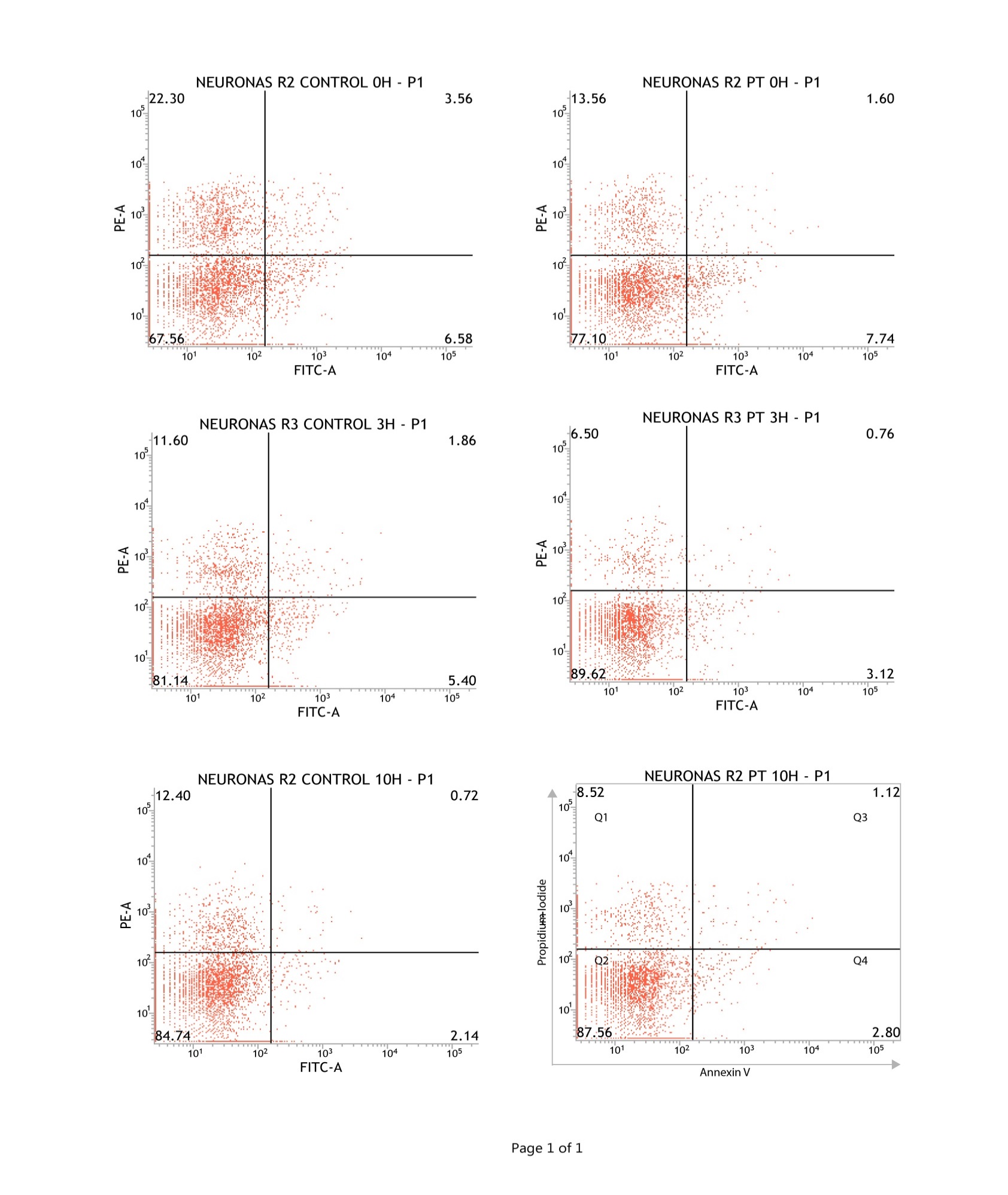

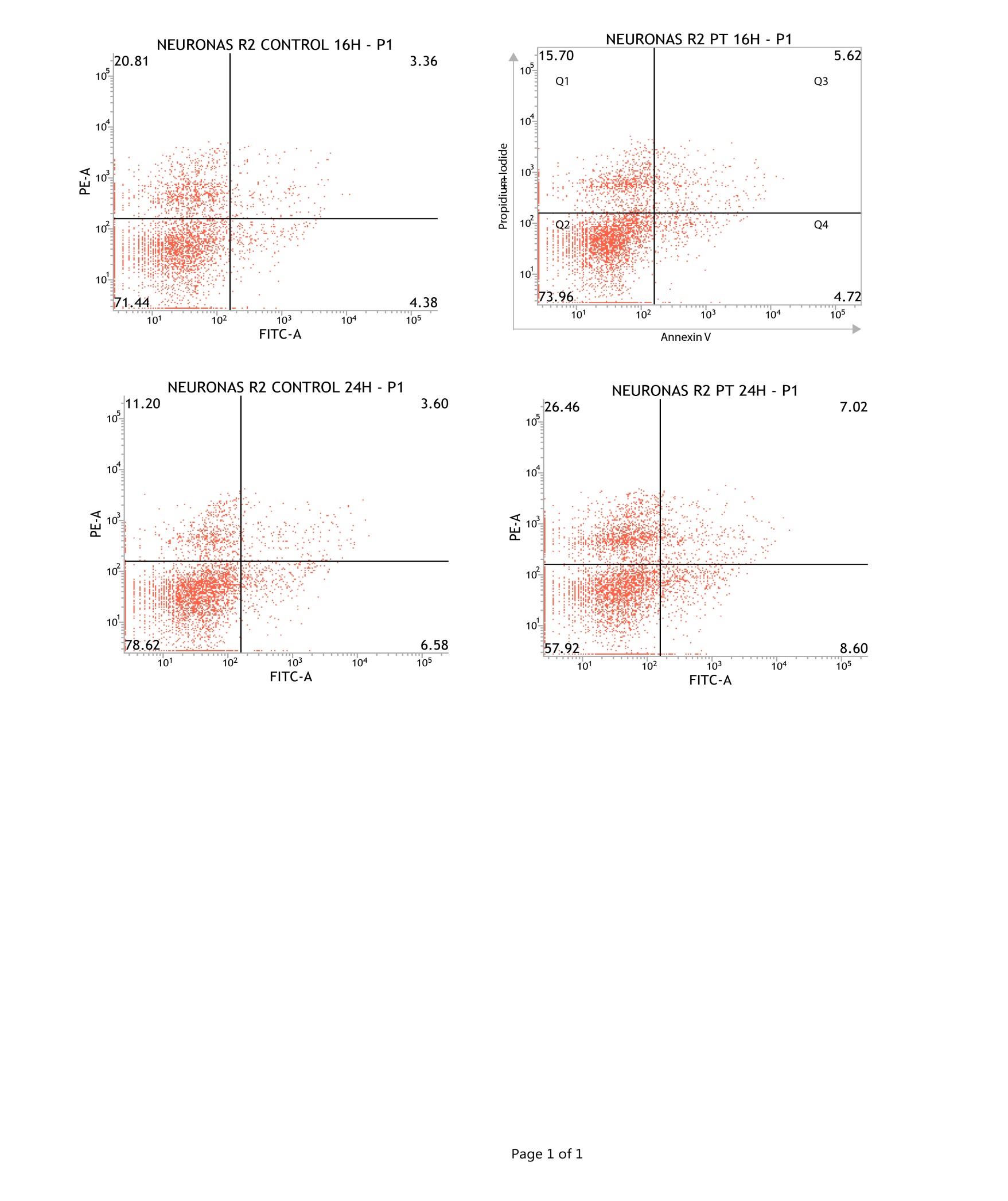
**

**pT 24 h**

**
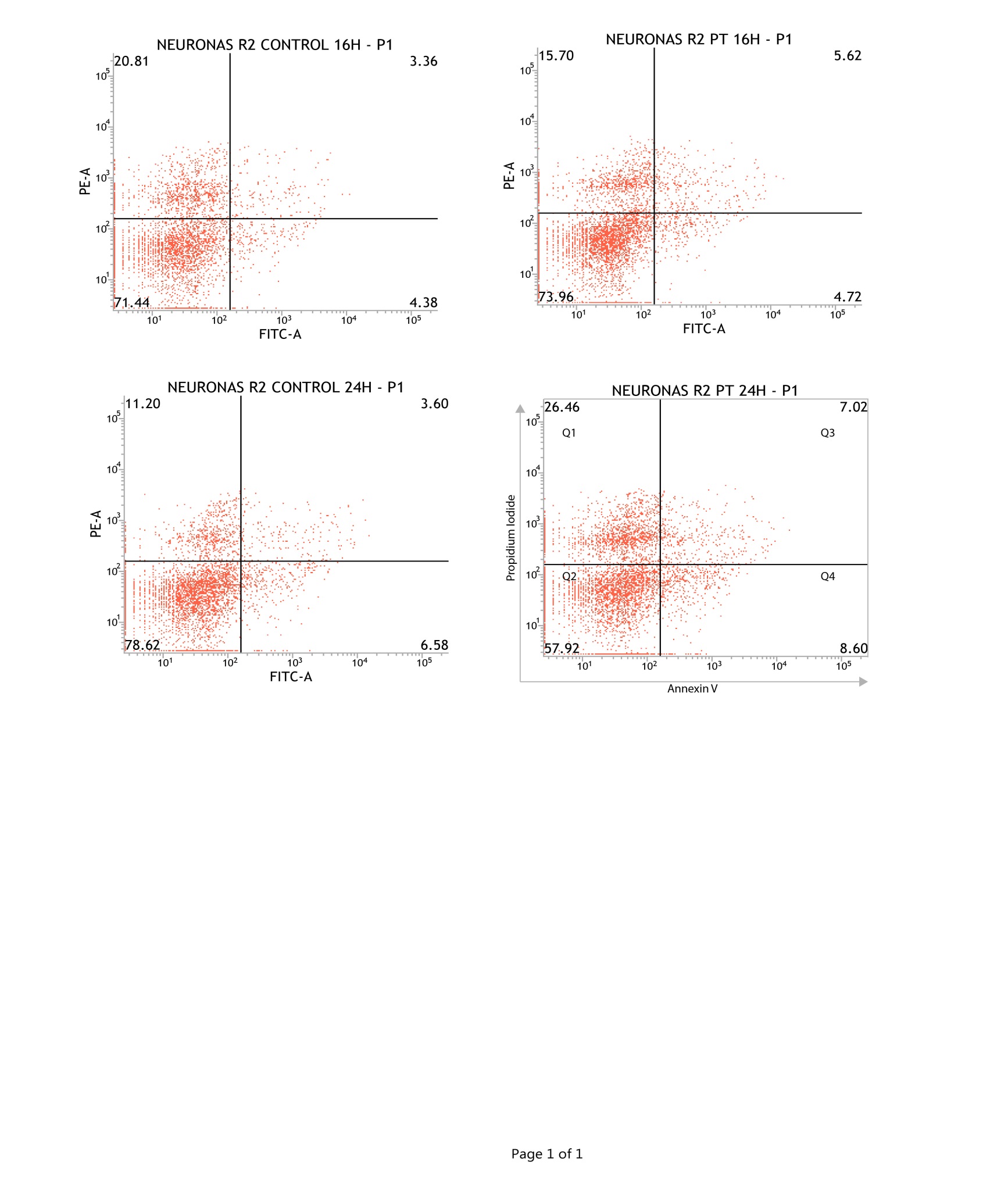
**

**Supplementary Figure 2 |** Representative assays of flow cytometry analysis for apoptosis in neurons after treatment with proTAME for 0, 4, 10, 16 or 24 hours.
